# Supplementary material for: A proline-rich motif on VGLUT1 reduces synaptic vesicle super-pool and spontaneous release frequency
Source: eLife. 2019 Oct 30;8:e50401. doi: 10.7554/eLife.50401 (PMC6861006; doi:10.7554/eLife.50401)
Supplement: Supplementary file 1. [file elife-50401-supp1.docx]

Table 1: Statistic of FRAP experiments

| **Experiment** | **Genotype/Mutant** | ***N/n*** | ***F* ratio (*F* test)** | ***P* (*F* test)** |
| --- | --- | --- | --- | --- |
| *vglut1^+/+^*  *vs. vglut1^-/-^*  (Fig. 1C) | Vglut1^+/+^ | 8/27 | 19.32 | <0.0001 |
|  | Vglut1^-/-^ | 11/31 |  |  |
| VGLUT1 rescue  vs.  VGLUT1 overexpression  (Fig. 1E) | VGLUT1 Rescue | 3/15 | 9.467 | <0.0001 |
|  | VGLUT1 Overexpression | 4/14 |  |  |
| sVGLUT1 *vs.* VGLUT1 *vs.* *vglut1^-/-^*  (Fig. 3B) | sVGLUT1 | 10/36 | 1.235**^#^** | 0.2941**^#^** |
|  | VGLUT1 | 5/23 | / | / |
|  | *vglut1^-/-^* | 7/16 | 30.25**^#^** | <0.0001**^#^** |
| Sham *vs.* SH3 mutants  (Fig. 5C – FRAP curves not shown) | mCerulean3-sham | 4/20 | / | / |
|  | mCerulean3-Endo-SH3 | 5/25 | 0.9942* | 0.4201* |
|  | mCerulean3-Endo-SH3^E329K,S336K^ | 4/18 | 15.84* | <0.0001* |
|  | mCerulean3-ITSN1-SH3B | 5/25 | 7.427* | <0.0001* |

Note: ***N*** for culture number, ***n*** for synapse number. **^#^** *F* test with VGLUT1; * *F* test with mCerulean3-sham.

Table 2: Statistic of VGLUT1^P554A^ FRAP experiment (Fig. 4 A)

| **Mutant** | ***N/n*** | **Fast Half-life (s)** | **Amplitude of the fast component (%)** | **Slow Half-Life (min)** | **Amplitude of the slow component (%)** | **Amplitude of recovery (%)** | ***F* ratio (*F* test)** | ***P* (*F* test)** |
| --- | --- | --- | --- | --- | --- | --- | --- | --- |
| VGLUT1 | 5/21 | 28.5 | 17.2 | 31.1 | 50.9 | 68.1 | 19.64 | <0.0001 |
| VGLUT1^P554A^ | 5/22 | 46.9 | 19.7 | 23.5 | 58.7 | 78.36 |  |  |

Note: ***N*** for culture number, ***n*** for synapse number.

Table 3: Statistic of electrophysiology experiments

| **Experiment** | **Genotype/ Mutant** | ***N/n*** | **mEPSC Amplitude** | | **mEPSC frequency** | | **mEPSC Amplitude** | | **mIPSC frequency** | |
| --- | --- | --- | --- | --- | --- | --- | --- | --- | --- | --- |
|  |  |  | **(pA) ± SEM** | ***P***  **(*t* test / one way ANOVA)** | **Hz ± SEM** | ***P***  **(t test / Mann-Whitney test)** | **(pA) ± SEM** | ***P***  **(*t* test / one way ANOVA)** | **Hz ± SEM** | ***P***  **(Mann-Whitney test)** |
| VGLUT1  *vs.*  sVGLUT1  Fig. 3 C-E  Fig. S3 | VGLUT1 | 3/31 | 15.3 ± 1.15 | 0.011 | 3.51 ± 0.6 | 0.008 | 18.06 ± 1.07 | 0.70 | 4.08 ± 0.59 | 0.62 |
|  | sVGLUT1 | 3/56 | 13.12 ± 0.71 |  | 2.51 ± 0.58 |  | 17.69 ± 0.73 |  | 3.99 ± 0.52 |  |
| VGLUT1 PP rescue  Fig. 4 G-I  Fig. S4 | Sham | 3/53 | 12.73 ± 0.79 | *vs.* VGLUT1^P554A^: 0.02 | 2.14 ± 0.37 | / | 19.29 ± 1.34 | / | 6.21 ± 0.69 | / |
|  | VGLUT1 | 3/61 | 16.05 ± 1.05 | *vs.* sham: 0.001 | 5.53 ± 0.87 | / | 22.47 ± 1.13 | / | 7.28 ± 0.69 | / |
|  | VGLUT1^P554A^ | 3/53 | 15.59 ± 1.02 | *vs.* VGLUT1: 0.485 | 7.59 ± 0.97 | *vs.* VGLUT1: 0.031 | 19.24 ± 0.78 | *vs.*VGLUT1: 0.137 | 6.79 ± 0.63 | *vs.* VGLUT1: 0.781 |

Note: *N* for culture number, *n* for cell number.

Table 4: Statistic of time-lapse imaging experiments. Fig. 4C-E

| **Mutant** | **Cumulative trafficking fluorescence** | | **Unpaired *t* test** | | **SV trafficking Speed** | | **One-way ANOVA** | | |  |
| --- | --- | --- | --- | --- | --- | --- | --- | --- | --- | --- |
|  | ***N*** | **Average trafficking fluorescence** | ***t*** | ***P*** | ***n*** | **Average speed** | | ***F*** | ***P*** | |
| VGLUT1 | 5 | 1.206 ± 0.0143 | / | / | 1145 | 1.564 ± 0.02692 | | 2.431 | 0.0634 | |
| VGLUT1^P554A^ | 4 | 1.508 ± 0.0948 | 3.564* | 0.0092* | 715 | 1.536 ± 0.02860 | |  |  |  |
| VGLUT1^S540A^ | 4 | 1.204 ± 0.0119 | 0.1351* | 0.8963* | 645 | 1.460 ± 0.02942 | |  |  |  |

Note: *N* for culture number, *n* for trafficking element number. * *vs.* VGLUT1.

Table 5: Statistics of Fluorescence recovery at 68 min for SH3 domains competitions. Fig. 5C

| **Mutant** | **Fluorescence recovery after 68 minutes** | | | **Mann-Whitney test** |
| --- | --- | --- | --- | --- |
|  | ***N*** | ***n*** | **Average fluorescence** | ***P*** |
| sham | 4 | 20 | 0.5346 ± 0.04128 | / # |
| Endo-SH3^WT^ | 5 | 25 | 0.6071 ± 0.03705 | 0.141 # |
| Endo-SH3^E329K,S336K^ | 4 | 18 | 0.7323 ± 0.04909 | 0.005 # |
| ITSN1-SH3B | 5 | 25 | 0.6894 ± 0.04036 | 0.014 # |

Note: *N* for culture number. *n* for synapse number. # *vs.* sham.

Table 6: Statistic of VGLUT2 FRAP experiments. Fig. S1

| **Experiment** | **Genotype/Mutant** | ***N/n*** | ***F* ratio (*F* test)** | ***P* (*F* test)** |
| --- | --- | --- | --- | --- |
| VGLUT1 *vs.* VGLUT2  rescue  (Fig. S1) | VGLUT1 | 7/24 | 19.27 | <0.0001 |
|  | VGLUT2 | 7/20 |  |  |

Note: *N* for culture number. *n* for synapse number.
